# Immediate and Long-Term Results of Coronary Angioplasty in Patients Aged 80 Years and Older

**DOI:** 10.4061/2010/263685

**Published:** 2010-06-20

**Authors:** Bo Chen, Dingguo Zhang, Tiebing Zhu, Liansheng Wang, Chunjian Li, Hui Wang, Fumin Zhang, Kejiang Cao, Wenzhu Ma, Zhijian Yang

**Affiliations:** ^1^Department of Cardiovascular Medicine, The First Affiliated Hospital of Nanjing Medical University, Nanjing, Jiangsu 210029, China; ^2^The Research Center for Bone And Stem Cells, Nanjing Medical University, Nanjing, Jiangsu 210029, China

## Abstract

*Objectives*. To observe the short- and long-term outcomes after percutaneous coronary intervention (PCI) in octogenarians (>80 y.o.) at our institution. *Method*. All octogenarians who underwent PCI during the study period were retrospectively retrieved from our database and clinically followed. Major adverse cardiac (and cerebral) events (MAC(C)E) was considered as primary outcome. *Results*. From January 2003 to December 2007, 140 octogenarians (mean age: 85±3 y.o., 79% of male) underwent PCI and were clinically followed 14±11 months. Procedural success was obtained in 100 percent of patients with single vessel disease, in 96 percent of patients with double vessel disease, and in 75 percent of patients with triple vessel disease. In-hospital, 30 days, and one year MACE rates were 5%, 5%, and 10.7%, respectively. Impaired left ventricular (LV) ejection fraction (hazard ratio (HR) = 0.909, 95% confidence interval (CI) = 0.856 to 0.964, *P* = .002), diabetes mellitus (HR = 5.792, 95%  CI = 1.785 to 18.796, *P* = .003), and low GFR (HR = 2.943, 95%  CI = 1.161, to 7.464, *P* = .023) were independently associated with an increase risk of MACE at long-term followup. *Conclusion*. Coronary angiography can be successfully performed in elderly patients with single and double vessel disease. The results in triple vessel disease are encouraging. Low LV function, diabetes, and impaired renal function increase the risk of long-term major adverse cardiac events.

## 1. Introduction

The proportion of older persons is increasing rapidly throughout the world, which projected to more than double worldwide over the next half century [1]. It is not surprising that more and more elderly individuals are at increased risk of coronary heart disease (CHD) and account for a majority of CHD deaths. Data showed nearly 83% of all CHD deaths and 88% of all stroke deaths are experienced by persons aged 65 years and older [1]. As a consequence, cardiologists are now encountering increasing numbers of highly symptomatic elderly patients with serious cardiac disease that warrants treatment. Advanced patient age, however, may often make attending physicians reluctant to pursue the necessary intervention of surgery or angioplasty. Controversy exists as to whether the considerable proportion of health care resources expended on the growing minority of very elderly patients represents a cost-effective approach in an attempt to maintain life at a meaningful level. Coronary artery bypass surgery improves long-term survival, but is associated with a high risk of perioperative morbidity and mortality. Percutaneous coronary intervention (PCI) is a less invasive procedure and could be an effective alternative in these elderly patients. However, the elderly patient has more advanced coronary artery disease and more calcified, or even rigid and tortuous lesions. This makes coronary angioplasty technically more difficult to perform and may lead to less satisfactory results [2]. At the same time, previous reports have suggested that PCI in elderly patients is associated with a higher complication rate [3–6]. It should be notice that some of these large studies referred to [6, 7] have the limitation of being multicenter registries, where data are self-reported and postinterventional complications could have been upreported. A recent systematic review and meta-analysis to evaluate the clinical outcome of patients aged 80 years and older undergoing coronary revascularization indicated clinical outcomes were similar for patients undergoing PCI and CABG despite higher preprocedural risk among patients undergoing PCI [8]. In this observational study, we report the clinical outcomes of coronary angioplasties between 2003 and 2007 for patients of 80 and older in the daily clinical practice of a single center catheterization lab.

## 2. Methods

### 2.1. Characteristics

There are 3,000 patients who underwent PCI since January 2003 till June 2007 at our institution. The retrospective data of 140 consecutive patients aged 80 or more with stable, chronic coronary heart disease were analyzed. The following characteristics were analyzed: clinical presentation, previous cardiac history, risk factor profile, extent of coronary disease, and left ventricular ejection fraction.

### 2.2. Procedure

Percutaneous transluminal coronary angioplasty (PTCA) was performed using standard technique and a steerable catheter system. PTCA was attempted on major coronary arteries which had ⩾70 percent narrowing diameter. Major arteries were defined as the left anterior descending and its large diagonal branches, the left circumflex and its large obtuse marginal branches, and a balanced or dominant right coronary artery. Patients who had a chronic total occlusion in one major coronary artery or those with poor left ventricular function were excluded. However, only patients with chronic total occlusion with favorable angiographic characteristics had PTCA attempted.

Generally, our strategy has been to attempt angioplasty of all significant stenoses during a single procedure whenever possible. However, in some patients, PTCA was staged as multiple procedures due to the unstable hemodynamic condition of the patients or the excessive administration of contrast.

If initially the patients' condition was hemodynamically unstable, only the culprit lesion was dilated. The culprit lesion was determined on the basis of coronary angiographic findings, electrocardiographic changes, and regional ventricular function. All patients were treated with aspirin 300 mg orally as the loading dosage and then 100 mg daily. Clopidogrel 300 mg loading dose, followed by 75 mg per day was administered after stent placement for at least 9 month. In the catherization laboratory, all patients were given 10000 units of heparin at the beginning of the procedure and additional heparin was given if the procedure was prolonged. After successful PTCA, low weight heparin was used for 3 to 5 days 100 U/kg body weight routinely.

### 2.3. Definitions

Multivessel disease was defined as a stenosis of ≥70 percent in at least two major vessels. Procedural success was defined as a reduction to residual stenosis of <40% by balloon angioplasty or successful stent deployment at the desired position with a residual stenosis <20% by visual assessment in multiple projections. Clinical success was defined as procedural success plus the absence of major adverse cardiac events (MACE) while in hospital for the index PCI—that is, myocardial infarction, the need for repeated revascularization, coronary artery bypass grafting (CABG), or death.

Complete revascularization was defined as successful dilation of all initial stenosis ≥70 percent. Incomplete revascularization was considered to be a successful angioplasty of one or more stenoses but with one or more residual stenoses of 70 percent remaining.

### 2.4. Followup

Clinical followup was obtained when patients were seen at the outpatient clinic or by direct telephone interview. Adjunctive information was obtained from the referring physician. Followup information included the following: current angina status, history of myocardial infarction since the initial angioplasty procedure, need for additional revascularization procedure (bypass surgery or repeated angioplasty), and death (cardiac or noncardiac). Furthermore, activity status was assessed by the patients' subjective impression when compared with activities prior to PTCA.

### 2.5. Statistical Analysis

Continuous variables are presented as mean  ±  SD. A multivariable Cox proportional hazard model was used to assess the predictors of MAGE after PCI. Variables included in the multivariate model were patient related factors. A *P*-value <.05 was considered significant.

## 3. Results

### 3.1. Clinical and Angiographic Characteristics

One hundred and forty consecutive patients > 80 years old (110 men), with a median age of 85 years (range 80–93 years), underwent intervention during the study period. Baseline clinical characteristics are shown in Table 1. Seventy eight of 140 of patients had multivessel disease, 32 had three vessel coronary disease, 46 two vessel coronary disease, and 62 single vessel coronary disease. Left ventricular fraction ⩽40 percent was present in 45 (32 percent) patients.

### 3.2. Initial Results

A total of 246 lesions were treated. Procedural success was obtained in 100 percent of patients with single vessel disease, in 96 percent of patients with double vessel disease, and in 75 percent of patients with triple vessel disease. Four total coronary occlusions were not attempted. The strategy for revascularization was PTCA alone in 7 patients, PTCA and stenting in 126 patients and direct stenting in 3 patients. 

Technical success was obtained in 126 patients with the success rate highest in patients with single vessel disease and lowest in patients with triple vessel disease. In patients with single vessel disease, complete revascularization was obtained in 100 percent. However, because of old total coronary occlusion or diffuse of disease in another artery, 69 percent of patients achieved complete revascularization with triple vessel disease, and 93 percent with two vessel disease.

*β*-blockers, angiotensin converting enzyme inhibitors/angiotensin II receptor blockers, and statin agents were administrated to all patients after PTCA if no contradiction existed.

### 3.3. Complications

In-hospital MACE rate was 7/140 (Table 1). The complication rate was highest among those patients who presented with severe coronary artery disease. The median time from procedure to discharge was 6 (3–25) days. Major hemorrhagic complications did not occur in any of the patients before discharge. None of the patients developed stroke, whereas two patients developed transient worsening renal dysfunction that resolved without the need for dialysis.

### 3.4. Late Results

Follow-up information was obtained on 97% patients. The mean follow-up time was 14 ± 11 months (range 3 to 45 months). The 30-day and one-year MACE rate was 5.0% and 10.7%, respectively (Table 2). The cause of death was definite myocardial infarction in six patients, congestive cardiac failure in 2 patients, and noncardiac death in 1 patient. In patients who died within 30 days of the procedure, five of six had had a recent myocardial, ejection fraction was <40% in four of six patients, and PCI had failed in two patients. 

Sixty four percent patients were subjectively improved at either the same or higher level of activity. Either within patients with clinical success or within patients without clinical improvement, the cardiac events were more common among patients with three-vessel disease or poor left ventricular function. Two patients had nondisabling stroke during followup, and one patient had a nonfatal gastrointestinal hemorrhage. 

Multivariate Cox proportional hazards analysis identified impaired LVEF (hazard ratio (HR) = 0.909, 95% confidence interval (CI) = 0.856 to 0.964, *P* = .002), diabetes mellitus (HR = 5.792, 95%  CI = 1.785 to 18.796, *P* = .003), and low GFR (<60 cc/min/1.73 m^2^) (HR = 2.943, 95%  CI = 1.161 to 7.464, *P* = .023) to be independently associated with an increased risk of MACE (Table 3).

### 3.5. Discussion

Recently, there has been a marked aging of the population, and concurrently, the treatment strategy for coronary heart disease in elderly patients has become more aggressive [1], especially in developing countries, such as China [4, 7, 9–11]. This is because not only these patients' life span has been prolonged but also there have been improvements in medical treatment in elderly patients. However, medical treatment is not always able to control symptoms and is sometimes poorly tolerated. In specific high risk subsets, medical therapy alone is not able to improve. PCI has been utilized for coronary revascularization for 30 years since the first application by Andreas Gruentzig in Zurich in 1977 [12]. Previous reports showed that these patients are at a high-risk because the major cardiac complications are higher than in younger patients. The elderly patients usually have more severe symptoms [13]. They frequently present with acute coronary syndrome. There are more patients with multiple-vessel diseases and more calcification on the coronary lesions and they are more commonly with comorbid conditions [14, 15]. Compared with younger patients, the elderly patients usually received incomplete revascularization. However, complete revascularization may not be necessary if the primary goal is to achieve symptomatic relief and coronary angioplasty can be performed safely and provides good symptomatic relief and favorable long-term outcome in old people over 70 years [16]. Furthermore, nowadays, in the era of stenting, multivessel disease and complex coronary anatomy, such as, chronic total occlusion, bifurcation lesions, heavily calcified lesions, orifice lesions, coronary graft lesions, left main stem lesions and smaller vessels are all candidate targets for intervention when indicated. Poor general conditions, such as acute myocardial infarction, heart failure or even cardiogenic shock are also proved to gain benefits from the interventions [17].

Our data showed that PTCA and stent can be done with high clinical success in elderly patients. Furthermore, in patients with multivessel disease, complete revascularization is still very high. The most common reasons for incomplete revascularization were long-term total occlusions and diffuse disease. In accordance with the literature, for patients at high risk in the presence of multivessel disease and low left ejection fraction, clinical success varies from 57 percent to 93 percent, and the inhospital mortality rate varies from 0.9 percent to 19 percent. These variabilities are probably due to patient selection and different populations studied. Recent reports on PTCA in patients with multivessel disease show that elderly patients frequently have incomplete revascularization and a higher rate of major cardiac complications because of the presence of severe coronary disease and/or low left ejection fraction. However, the use of the stent could possibly reduce the complication rate. Hussain et al. [18] showed that PTCA with stent placement can be performed in octogenarians with a high rate of clinical and angiographic success with an acceptable range of morbidity and mortality, and favorable long term (two year) outcome. In this study, the extracardiac complications which are frequent after surgical revascularization were limited, and yet more important is the fact that no patient suffered a cerebrovascular accident. 

Followup results were encouraging. For 84 percent of patients in our study who had clinical success after PTCA, the long-term results continued to be beneficial. Mortality was relatively low and usually involved those patients with triple vessel disease, low ejection fraction, and incomplete revascularization. Jeroudi et al. [19] showed that survival at one year was 81 percent and at three years, 80 percent. On the other hand, Imburgia et al. [20] reported the lowest late success rate. Of 26 patients who achieved clinical success, only 11 had improved at followup. The different long-term results can be explained by the baseline clinical variables pre-PTCA and the various degrees of revascularization obtained. Some authors assert that patients with incomplete revascularization obtained by PTCA have a higher rate of symptoms and coronary bypass at followup than patients who have had complete revascularization [21]. We identified low LV function, diabetes and impaired renal function as independent predictors of unfavorable outcome at long-term followup. Bell et al. reported that survival of patients with multivessel disease is influenced by the baseline clinical variables but not the degree of revascularization [22]. The CASS study [23] showed that survival of elderly patients was influenced by both the left ejection fraction and also the associated medical disease. Only a prospective randomized study will answer the question of what treatment is best in selected patients.

It can be hypothesized that if long-term outcome depends on baseline clinical characteristics rather than degree of revascularization, PTCA and stent can be a valid therapeutic alternative even if the degree of revascularization is lower.

#### 3.5.1. Study Limitations

Our data come from a relatively small sample size and were obtained in a single center. No comparison of the results in this older population to the large volume of younger patients was obtained. However, some important studies have demonstrated that this group of elder patients has higher in-hospital mortality when comparing with younger patients [12, 22].

## 4. Conclusions

Percutaneous coronary angioplasty is a valid therapeutic alternative in elderly patients with coronary artery disease. In selected patients with single and double vessel disease, the short- and long-term outcome is very favorable. In patients with triple vessel disease, the short-term results are less favorable. However, these patients with triple vessel disease and complete revascularization by PTCA have good long-term outcome. Low LV function, diabetes, and impaired renal function increase the risk of long-term major adverse cardiac events.

## Figures and Tables

**Table 1:** Baseline clinical characters.

Age (years) (mean ± SD)	85 ± 3
Gender (*n*, % male)	110 (79%)
BMI (kg/m^2^) (mean ± SD)	24.4 ± 2.4
History of (*n*, %)	
Hypertension	129 (92%)
Diabetes mellitus	53 (38%)
Cigarette smoking	15 (11%)
Hyperlipidaemia	32 (23%)
Stroke	11 (8%)
Myocardial infarction	20 (14%)
Angina pectoris	135 (96%)
Medications (*n*, %)	
Aspirin	138 (99%)
Clopidogrel	6 (4%)
*β*-blocker	135 (96%)
ACEI/ARB	140 (100%)
Digitalis	10 (7%)
Diuretics	120 (86%)
Calcium channel blockers	4 (3%)
Nitrates	128 (91%)
Statin	140 (100%)
eGFR (ml/min) (mean ± SD)	65.7 ± 14.6
Fasting lipid values	
Total cholesterol (mg/dL) (mean ± SD)	192.2 ± 27.4
Triglycerides (mg/dL) (mean ± SD)	144.9 ± 31.5
HDL (mg/dL) (mean ± SD)	42.9 ± 7.9
LDL (mg/dL) (mean ± SD)	120.3 ± 27.2
CCS(II/III/IV) (*n*,%)	12/82/5 (8%/59%/4%)
LVEF mean (%) (mean ± SD)	46 ± 10 (20–70)
<40%	45 (32%)

ACEI/ARB, angiotensin converting enzyme inhibitor/angiotensin II receptor blocker; BMI, body mass index; eGFR, estimated glomerular filtration rate; SD, standard deviation.

**Table 2:** Major adverse cardiac events.

	In-hospital *n* (%)	30 days *n* (%)	1 year *n* (%)
Death	4 (2.9)	2 (1.4)	1 (0.7)
Myocardial infarction	0 (0)	2 (1.4)	1 (0.7)
Repeat PCI	0 (0)	1 (0.7)	13 (9.3)
CABG	3 (2.1)	2 (1.4)	0 (0)
Total	7 (5.0)	7 (5.0)	15 (10.7)

CABG: Coronary artery bypass graft; PCI: Percutaneous coronary intervention.

**Table 3:** Multivariate Predictors of MACE after PCI.

Predictors of MACE	HR	95% CI	*P* value
LV Ejection fraction	0.909	0.856–0.964	.002
Diabetes mellitus	5.792	1.785–18.796	.003
eGFR	2.943	1.161–7.464	.023

CI, confidence interval; eGFR, estimated glomerular filtration rate; HR, hazard ratio; LV, left ventricular; MACE, major adverse cardiac events.
